# Inter‐country distancing, globalisation and the coronavirus pandemic

**DOI:** 10.1111/twec.12969

**Published:** 2020-06-09

**Authors:** Klaus F. Zimmermann, Gokhan Karabulut, Mehmet Huseyin Bilgin, Asli Cansin Doker

**Affiliations:** ^1^ Global Labor Organization, and Centre for Economic Policy Research UNU‐MERIT & Maastricht University Maastricht The Netherlands; ^2^ GLO Istanbul University Istanbul Turkey; ^3^ GLO Istanbul Medeniyet University Istanbul Turkey; ^4^ GLO Erzincan Binali Yildirim University Erzincan Turkey

**Keywords:** coronavirus, COVID‐19, globalisation, inter‐country distancing, pandemic

## Abstract

Originating in China, the coronavirus has reached the world at different speeds and levels of strength. This paper provides an initial understanding of some driving factors and their consequences. Since transmission requires people, the human factor behind globalisation is essential. Globalisation, a major force behind global well‐being and equality, is highly associated with this factor. The analysis investigates the impact globalisation has on the speed of initial transmission to a country and on the scale of initial infections in the context of other driving factors. Our cross‐country analysis finds that measures of globalisation are positively related to the spread of the virus, both in speed and in scale. However, the study also finds that globalised countries are better equipped to keep fatality rates low. The conclusion is not to reduce globalisation to avoid pandemics, but to better monitor the human factor at the outbreak and mobilise collaboration forces to curtail diseases.

## 1 INTRODUCTION

In response to the coronavirus pandemic against which there is currently no proven vaccine or drug treatment, human mobility between and within countries in general has been stopped on a temporary basis since April 2020. The lockdown of economies and suspension of free mobility were justified by a rapid transmission of the virus through the human factor of globalisation, namely personal interactions. Social distancing at the individual level was complemented by *inter‐country distancing*. The development is marked by a number of disturbing factors: global termination of travel mostly via national policy responses; attacks on global organisations such as the World Health Organization; the conflict between states over pharmaceutical tools and the support of medical research companies; and the de facto absence of leadership from international organisations like the European Union or G20 in response to this crisis.

Powerful diseases can spread globally and generate pandemics that can end up seriously affecting almost all countries. It is important to understand the disease transition to be able to improve defence mechanisms, strengthen healthcare sectors, find a vaccine and intercept infection channels even if transmission cannot be stopped completely.

Globalisation is the final result of the division of work that creates welfare, but it might potentially facilitate the spread of infection. The process can have an impact on the spread of disease by many different channels including international trade, international tourism, international students, migration and transportation. Globalisation has been attacked as a 'cause' of this pandemic. Hence, we are interested in the initial impact it has on affected countries in terms of transmission speed and mortality consequences, conditioned on other driving factors.

The paper is organised as follows. Section 2 discusses relevant background knowledge on pandemics and their interaction with globalisation. Section 3 presents methodology and data, and section 4 reports empirical findings and robustness checks. Section 5 concludes.

## 2 PANDEMICS AND GLOBALISATION

Anti‐globalist arguments have a long tradition in the history of pandemics. The current coronavirus pandemic is already considered to be a major challenge to mankind, although not comparable to the *Black Death 1,346—1,353* in Europe (Benedictow, 2004) or the *1918—1920 Flu Pandemic* ('Spanish Flu'). Black Death is thought to have originated in Central or East Asia and spread to Europe via trade along the Silk Road, while Spanish Flu can be traced back to a US military personnel from Fort Riley, Kansas traveling with the US troops to Europe during World War I. Mankel et al. (2007) report 40 million deaths worldwide due to the *Flu Pandemic*, but estimates typically vary in the literature between 17 and 50 million. Black Death is reported to have resulted in 25–50 million casualties in Europe and about 75–200 million in Eurasia and North Africa. With over 170,000 deaths worldwide associated with the coronavirus so far, the current burden still seems comparatively small,^1^ yet the healthcare systems of some countries are already under substantial pressure. But given the likelihood of several mortality waves (the *Flu Pandemic* had three, with the second being the strongest by far) and the fact that we are just at the beginning of the pandemic, there is great uncertainty.

With no proven medical treatment or vaccine available, the current challenge is not so different from the *Flu Pandemic*. The only available short‐term options outside the healthcare sector are strategies of social and inter‐country distancing including lockdowns and border closures. The year 1918 marked the end of World War I, with many (mostly unfriendly) cross‐country human interactions. The world had been fairly globalised before World War I. In fact Flandreau, Flores, Clemens, and Khoudour‐Casteras (2010, see pp. 100–101, in particular figure 4.3) argue that characterising globalisation as trade openness, financial integration and international migration, the world was even more open than today for financial integration and (most important in our context) international migration.

Social and inter‐country distancing are concepts that are obviously in conflict with globalisation. But what do we know about how they work from the *Flu Pandemic* and the current *COVID‐19 coronavirus* experiences? Mankel et al. (2007) investigated non‐pharmaceutical interventions in 43 US cities from September 1918 to February 1919 to examine whether their timing, duration, and combination were linked to the observed city‐to‐city mortality variation. The interventions were studied under 3 major categories: (a) school closure, (b) cancellation of public gatherings, and (c) isolation and quarantine. Results strongly supported a negative association between the duration of non‐pharmaceutical interventions and mortality. According to Qiu, Chen, and Shi (2020) who studied responses to the coronavirus in China from January to February 2020, stringent quarantine, city lockdown and local public health measures significantly decreased the transmission rate. Outmigration from the outbreak source region (the city of Wuhan and Hubei province) showed a much stronger transmission factor to their destination regions compared to determinants like geographic proximity and economic conditions. Fang, Wang, and Yang (2020), Zhan, Tse, Fu, Lai, and Zhang (2020) and Zhang et al. (2020) also find that reducing human mobility mitigated the coronavirus transmission in China. Studies on other viruses have shown that the spread is faster during economic booms (Adda, 2016) and with trade growth (Adda, 2016, on influenza; Oster, 2012, on HIV). There may be also long‐term growth effects through changes of fertility (Chin & Wilson, 2018).

This research suggests social distancing within countries and more importantly distancing between countries early on, focusing on the human factor are crucial to avoid a pandemic or at least to contain it. Hence, strict monitoring of human mobility across borders (including their closure) may seem appropriate. In the face of the current coronavirus threat, would this require reducing globalisation in the future?

There were also anti‐globalist arguments during the more recent 2003 outbreak of SARS (severe acute respiratory syndrome) that started spreading to other countries from Hong Kong. At the time, the speed of transmission was so fast that a future pandemic seemed possible. Fears that originated in the affected countries at that time did not disappear with containment of the virus (Cheng, 2004). While several countries were affected, it was still possible to stop SARS before it became a pandemic (Chan‐Yeung & Xu, 2003). But it was the first international epidemic of the 21st century. During that period, the SARS epidemic also triggered an anti‐globalism discourse (So & Pun, 2004). Even the World Health Organization (WHO) stated that a new disease with wide‐ranging impact might appear soon in a world becoming more and more interconnected with cross‐boundary interactions becoming easier and more commonplace (WHO, 2003). However, they also report that globalisation might enable rapid information exchange between countries and a quicker response against a pandemic. With the COVID‐19 outbreak becoming a pandemic, similar anti‐globalist feelings have started to emerge (Legrain, 2020 and Oba, 2020). Many governments have limited the export of medical supplies and medicines (Evenett, 2020). These discussions may result in a more permanent negative effect on the globalisation process since the impact of Coronavirus on the world is much bigger than that of SARS. There was already a lively debate on globalisation underway which this may accelerate (James, 2002).

Since globalisation is not solely a political choice, but a phenomenon related to various factors such as transportation and technology (especially those that affect information flows, see Ozcan, 2018), as well as a matter of the optimal division of work, it seems to be an irreversible process. Countries with globally diversified production are much more resilient to all kinds of shocks. Issues traditionally considered to be of local concern are only now seen as globally relevant and to be addressed through global collaborations. Such collaborations are needed at the beginning of a pandemic in particular to manage human mobility, while capital movements and trade policies can remain liberal (Evenett, 2020).

## 3 METHODOLOGICAL APPROACH AND DATA

We are interested in the initial impact the pandemic has on affected countries in terms of transmission speed and mortality consequences. We neither model the evolution of the epidemic nor attempt to study the impact of health measures to contain the infection. We are only interested in understanding initial forces that drive the spread of the infection around the world. The value of such analysis is that it enables policymakers to better judge their options and the time constraints on action.

The *transmission speed* (TS) of the pandemic from country of origin (China) to another country is defined as

$$Transmission\;\;speed\;\left(\mathrm{TS}\right)\;=\;duration\;\;to\;\;reach\;\;country\;\;\left(D\right)\;\;times\;\;the\;\;infection\;\;rate\;\;\left(\mathrm{CP}\right),$$

whereas D is the *duration* (in days) between the outbreak in China^2^ and the first recorded case in a particular country (day gap), and CP is the *infection rate* defined as the number of confirmed COVID‐19 cases C divided by P, the respective population size:

$$Infection\;\;rate\;\;\left(\mathrm{CP}\right)\;\;=\;\;number\;\;of\;\;COVID-19\;\;cases\;\;divided\;\;by\;\;population\;\;size\;P.$$

As a major outcome variable, we measure the initial impact on mortality captured by the *case fatality rate* (CFR) defined in the epidemiology literature (Kelly & Cowling, 2013) as the proportion of deaths (M) from the disease divided by the number of confirmed infection cases C:

Case fatality ratio^3^ (CFR) = number of deaths (M) divided by the confirmed cases C.

Due to the non‐linear structure of the data,^4^ we analyse the variables linearised as ln TS, ln D, ln CP and ln CFR.^5^ We use the COVID‐19 data from the Johns Hopkins University Coronavirus Resource Center and will refer to the four variables as Coronavirus Variables in what follows. The data were collected for March 16, which is a few days after the global pandemic declaration on March 11, to avoid effects of government responses that could affect the data due to biological factors about two weeks later. The mortality data (M) are taken from April 6 assuming some delay between infections and deaths. The quality of the infection and mortality data is sometimes debated. However, Jelnov (2020) shows that the cross‐country correlation between the log of tests and log of reported cases (per capita) and correlation between log of reported cases and log of reported deaths (per capita) is high, suggesting reliability.

As discussed above, our key hypothesis is that the degree of globalisation reflects important channels that impact the time and size of initial infection across countries. Understanding this relationship is important to enable governments to better design and execute non‐pharmaceutical interventions. We measure globalisation using three different indices ('de facto', 'de jure' and 'overall') provided by the Swiss Federal Institute of Technology (KOF).^6^ The 'de jure' index concentrates on trade regulations, tax regime, investment restrictions, tourism and capital regulations, international treaties, tariffs and several other legal matters; the 'de facto' index measures actual amounts of trade, foreign investment, international tourism, international students, migration and capital movements; and the 'overall' index combines the two. The alternative measures may provide insights into the nature of the disease's relationship with globalisation and are useful for robustness checks. For instance, the 'de facto' measure of globalisation contains more information related to actual human mobility and should potentially have a larger effect on the transmission of the disease.

The baseline equation is:

(1) $$Coronavirus\;Variables_{i}=\gamma_{0}+\gamma_{1}Economic\;Globalization_{i}+\gamma_{1}X_{i}+\varepsilon_{i}$$

$X_{i}$ denotes the vector of controls and $\varepsilon_{i}$ is the error term in the country i. *Coronavirus Variables* are D, CP, TS or CFR, *Economic Globalization* is KOF over, KOF de facto or KOF de jure. Control variables are average temperature in March, the median age of the population, population age 65 and above as a percentage of the total population, distance in km between Beijing and the respective country's capital, a democracy index (Institutionalised Democracy Index), a 'Belt Country' dummy variable for the member countries of China's *One Belt One Road* project, and an index for government ideology with values 1 for right, 2 for moderate and 3 for left. We use the following variables in ln form to model the non‐linear relationship in the data and simplify interpretation: *Coronavirus Variables*, *Economic Globalization* variables, median age of the population, population with age 65 and above as a percentage of the total population, and distance from Beijing. The available dataset includes the 118 countries listed in the Appendix. Definitions and sources of all variables and their descriptive statistics are provided in Table 1. The data set contains 101 countries for the analysis of the non‐zero case fatality ratios.

**Table 1:** Summary statistics

Variables/Variable names in model	Definition	Data source	Mean	*SD*	Minimum	Maximum	Obs.
Transmission Speed/LnTS	Total number of Corona cases/population*day gap	Johns Hopkins University Center for Systems Science and Engineering (2020)	8.672	2.428	−14.322	−4.235	123
The Day Gap to First Case Wuhan/LnD	The difference between first case in Wuhan and first confirmed case by country	Johns Hopkins University Center for Systems Science and Engineering (2020)	3.993	0.375	3.091	4.330	123
Confirmed Cases per capita/LnCP	Total number of Corona cases/population	Johns Hopkins University Center for Systems Science and Engineering (2020)	−12.66	2.530	−18.399	−7.677	125
Case Fatality Ratio/LnCFR	Total Number of Death/Total Number of Corona case	Johns Hopkins University Center for Systems Science and Engineering (2020)	−0.932	1.488	−4.985	3.326	110
Economic Globalization (Overall)/Ln KOF‐over	Logarithmic Form	KOF: Gygli, Haelg, Potrafke, and Sturm (2019)	4.194	0.221	3.443	4.513	125
Economic Globalization (de facto)/Ln KOF‐de facto	Logarithmic Form	KOF: Gygli et al. (2019)	4.229	0.221	3.485	4.534	125
Economic Globalization (de jure)/Ln KOF‐de jure	Logarithmic Form	KOF: Gygli et al. (2019)	4.154	0.238	3.387	4.519	125
Gross Domestic Product per capita/Ln GDP‐per capita	Constant LCU	WDI: World Bank (2019)	11.508	2.332	5.697	17.477	123
Democracy Index/Democracy	Index from −77 to 10	Database of Political Institutions: World Bank (2019)	4,688	10,597	−77	10	122
Monthly Temperature/Temperature	Average March temperature for each country	World Bank API (2020)	16,14	10.276	−8.3	32.2	125
Age 65 and above Population (%)/Ln Age 65+	Population ages 65 and above as a percentage of the total population	World Bank (2019)	2.053	0.764	0.086	3.317	124
Government Ideology (Chief Executive's Party's Value)	Dummy Variable	Database of Political Institutions: Cruz, Keefer, and Scartascini (2018)	1.768	0.871	1	3	125
Distance to China (Beijing) By Km/Ln Distance	Distance between China and Country Capital	Google Earth Measurement (2020)	8.939	0.530	6.993	9.865	123
One Belt One Road Rotation/Belt Countries	Dummy Variable	Zhang and Fang (2020)	0.064	0.245	0	1	125
Median Age/Ln Median Age	Median Age of Population	United Nations (2019)	3.422	0.292	2.850	3.972	123

## 4 EMPIRICAL FINDINGS

**Figure 1:**
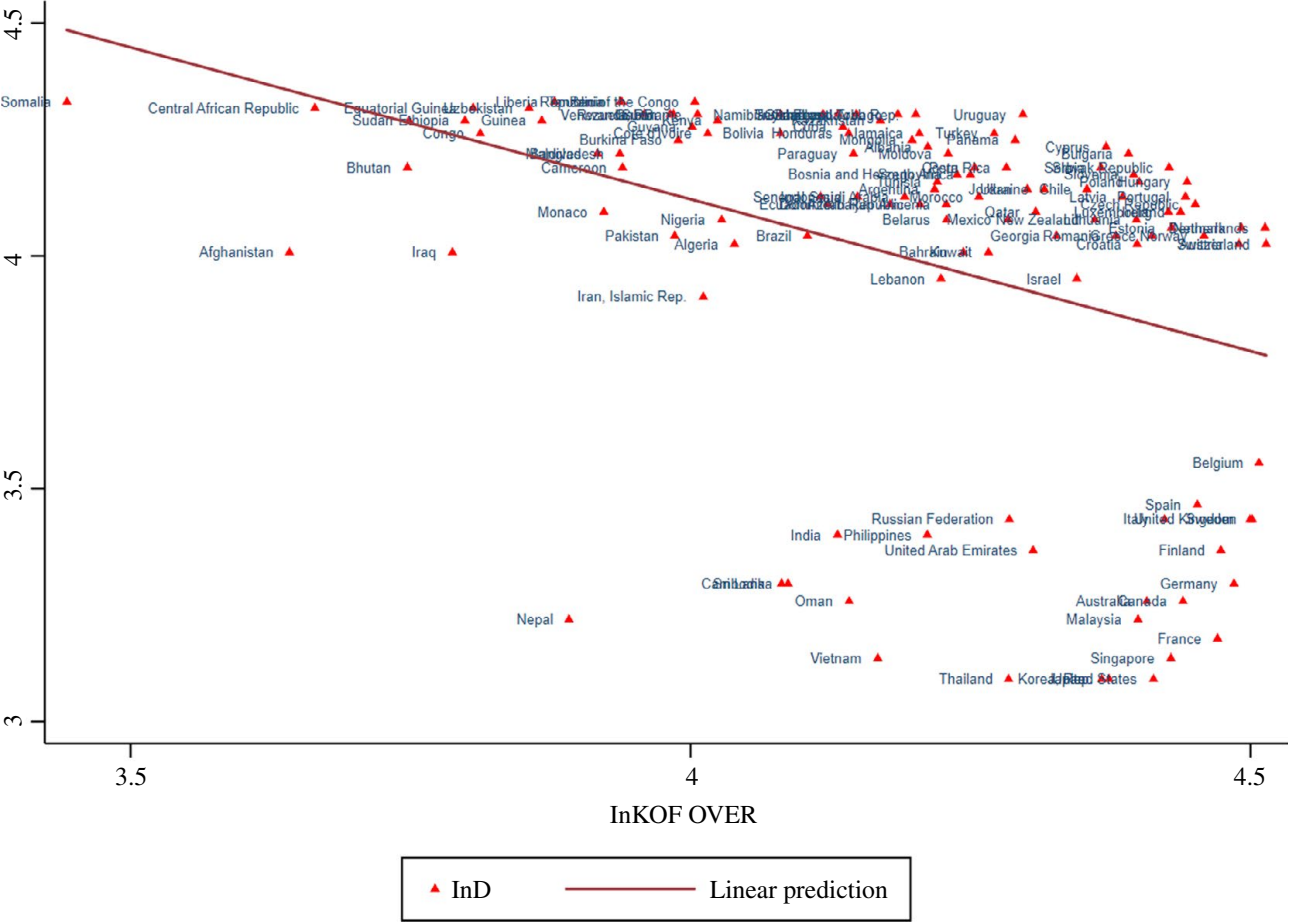
Globalisation and transmission duration (Ln KOF over and LnD) [Colour figure can be viewed at wileyonlinelibrary.com]

**Figure 2:**
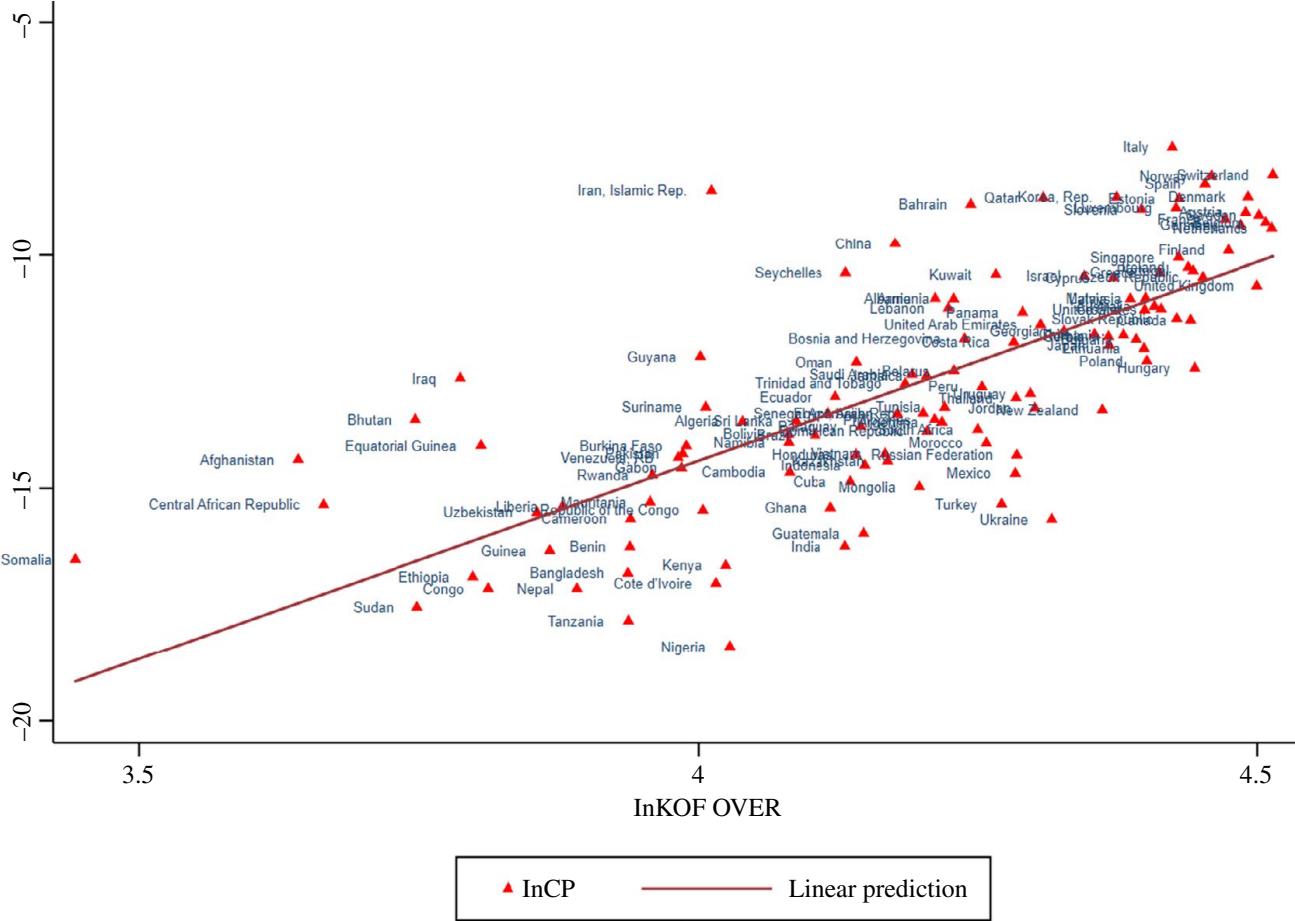
Globalisation and infection rate (Ln KOF over and LnCP) [Colour figure can be viewed at wileyonlinelibrary.com]

**Figure 3:**
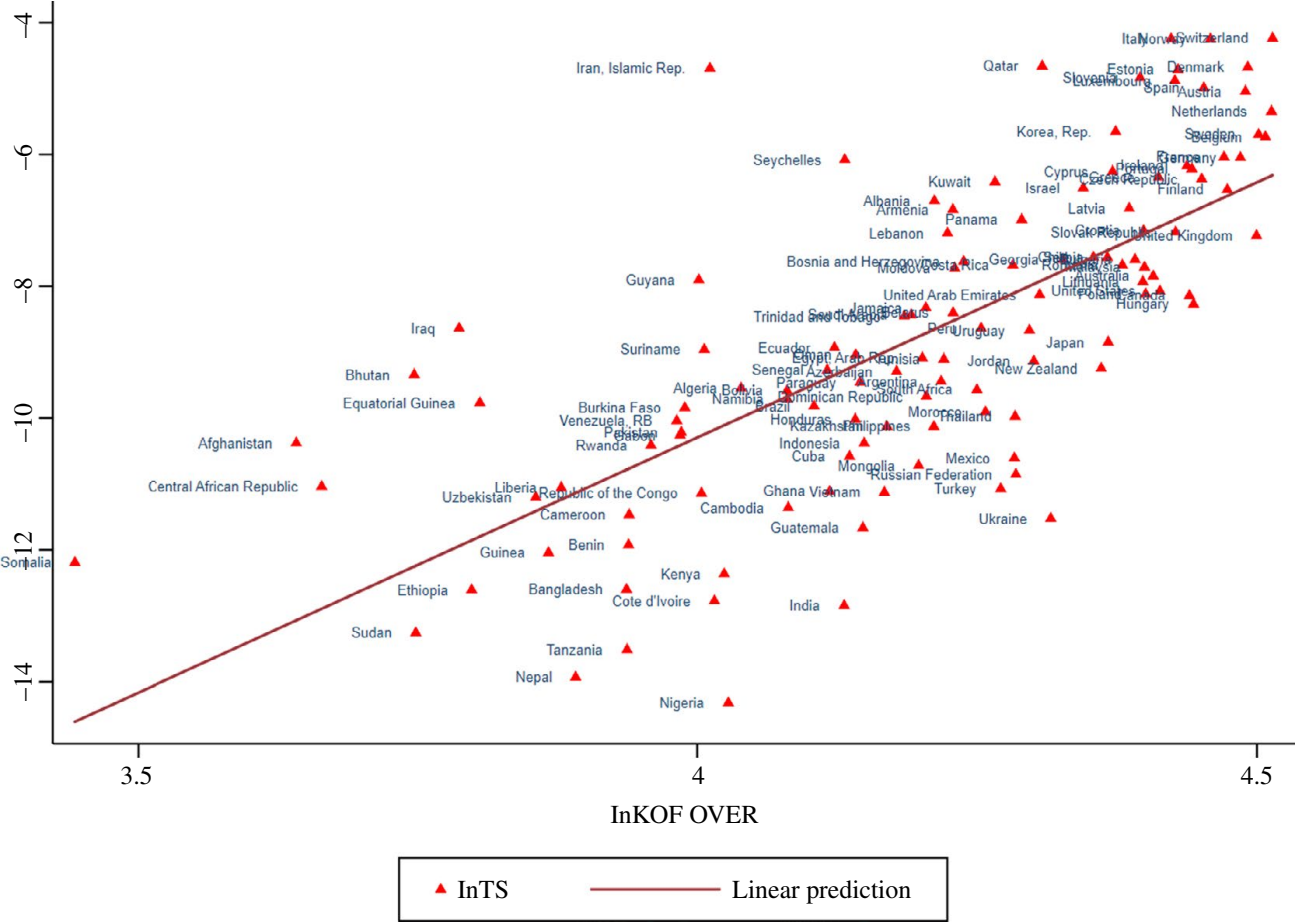
Globalisation and transmission speed (Ln KOF over and LnTS) [Colour figure can be viewed at wileyonlinelibrary.com]

**Figure 4:**
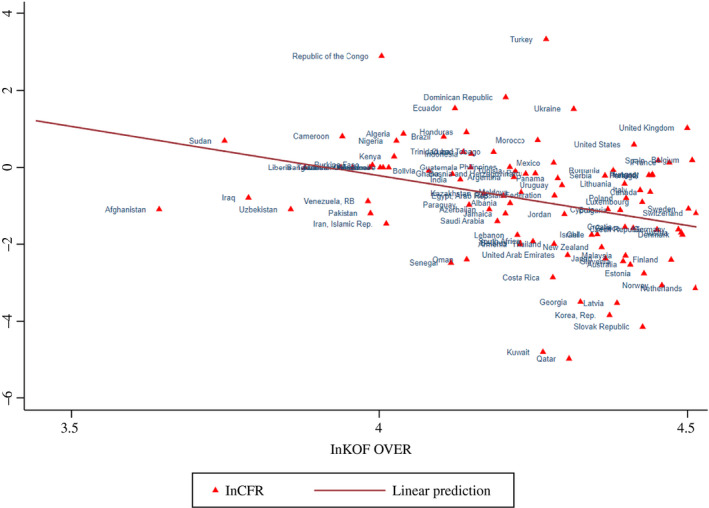
Globalisation and case fatality ratio (Ln KOF over and LnCFR) [Colour figure can be viewed at wileyonlinelibrary.com]

An initial illustration of the relationships between the *Coronavirus Variables* and *Economic Globalization* (KOF over) is provided in Figures 1, 2, 3, 4. In Figure 1, a negative relationship between ln D and ln KOF over can be observed. Several African countries and Afghanistan are clustered in the upper left of the figure. Although many Chinese workers are present in African *One Belt One Road* countries, they are known to live there fairly isolated. Most countries affiliate closely to the drawn line, showing a somewhat slower, but falling transition duration. A number of countries assemble in the lower right of the figure indicating that they are all fairly globalised with some located closely geographically (Nepal, Vietnam, Cambodia, India, Thailand, Malaysia, Philippines, Russia) and others are particularly global and developed (Italy, France, Germany, Belgium, the United States, South Korea, Australia, Canada and Spain). The other figures do not have such a clear separate cluster. Infections and globalisation are upward‐sloping: Figure 2 connects ln KOF over with ln CP, the logged infection rate. Clearly, above the line are Afghanistan, Iraq, Iran and Italy, and below are countries such as Tanzania, Nigeria, Turkey and Ukraine. It is very likely that the challenge is much more marked for developing countries in the longer run as soon as the spread is better measured and had more time to get into effect. Figure 3 deals with the transmission speed (ln T), which combines the previous two pictures confirming basically the relationship revealed by Figure 2.

Finally, Figure 4 shows that the case fatality ratio (ln CFR) declines with larger globalisation (ln KOF over) with clear outliers Congo and Turkey above and Kuwait and Qatar below the line. We argue that the fatality statistic we are using is largely reflecting the infection situation before the global pandemic declaration on March 11 and the lockdown decisions of many countries that were executed only step by step thereafter. Therefore, Figure 4 does not inform about country response strategies and their success, which would be much too early to judge at the time when this paper was completed. Nevertheless, there are clear differences among the globalised countries, indicating initial diverse policy stands: among others, the United Kingdom, the United States, Spain, Belgium and France are above the line, while Sweden is close to the line, and Finland, Norway, the Netherlands, South Korea and the Slovak Republic are below.

These core findings are confirmed by various regressions. Table 2 contains the OLS estimates of Equation (1) in four parts, each with the three alternative measures for globalisation as a robustness check. Globalised countries have consistently received the virus faster (D), with a higher infection rate (CP) and a higher transmission speed (TS), but also with a lower case fatality ratio (CFR). Transmission speed and both of its components D and CP exhibit estimates that all have 1% significance with coefficient sizes for KOF de jure that are somewhat smaller in absolute terms. This is plausible since the KOF de facto measure is more closely related to actual human mobility. The findings for the case fatality ratio confirm this insight: globalised economies seem to be more competitive in managing the infection, and the significance and size of the effect here comes primarily through KOF de facto, stressing the importance of human mobility. The KOF de facto coefficient is significant at 5% and much larger in absolute terms than the KOF de jure coefficient, which is significant only at 10%.

**Table 2:** OLS results

Regressors	LnD			LnCP			LnTS			LnCFR		
	(I)	(II)	(III)	(IV)	(V)	(VI)	(VII)	(VIII)	(IX)	(X)	(XI)	(XII)
Constant	3.740***	3.801***	3.324***	−48.564***	−48.770***	−45.837***	−44.823***	−44.968***	−42.513***	19.059***	20.998***	16.295***
	(1.111)	(1.077)	(1.113)	(6.071)	(5.525)	(6.369)	(6.193)	(5.987)	(6.304)	(4.655)	(5.577)	(3.968)
Ln KOF over	−0.715***	–	–	4.735***	–	–	4.019***	–	–	−2.372**	–	–
	(0.188)			(1.372)			(1.330)			(1.168)		
Ln KOF de facto	–	−0.718***	–	–	4.649***	–	–	3.931***	–	–	−2.685**	–
		(0.200)			(1.476)			(1.451)			(1.517)	
Ln KOF de jure	–	–	−0. 515***	–	–	3.427***	–	–	2.912***	–	–	−1.536*
			(0. 161)			(1.204)			(1.132)			(0.865)
Temperature	−0.015***	−0.016***	−0.014***	−0.009	−0. 005	−0.013	−0.024	−0.021	−0.027	0.003	0.0019	0.004
	(0.005)	(0.006)	(0.005)	(0.023)	(0.022)	(0.024)	(0.023)	(0.022)	(0.023)	(0.019)	(0.019)	(0.019)
Ln age 65+	−0.085	−0.063	−0.104	−0.765*	−0.910*	−0.642*	−0.851*	−0.973*	−0.746*	1.754***	1.658***	1.453***
	(0.094)	(0.096)	(0.095)	(0.404)	(0.419)	(0.410)	(0.449)	(0.461)	(0.450)	(0.394)	(0.415)	(0.386)
Ln median age	−0.093	−0.162	−0.159	5.410***	5.910***	5.829***	5.308***	5.748***	5.670***	−3.809***	−4.032***	−3.999***
	(0.231)	(0.232)	(0.236)	(1.532)	(1.554)	(1.489)	(1.572)	(1.589)	(1.526)	(1.253)	(1.267)	(1.257)
Ln distance	0.449***	0.063***	0.429***	−0.006	−0.177	0.077	0.416	0.291	0.506	–	–	–
	(0.060)	(0.065)	(0.062)	(0.363)	(0.378)	(0.377)	(0.373)	(0.373)	(0.378)			
Democracy	0.002*	0.001	0.002	−0.006	−0.007	−0.005	−0.005	−0.004	−0.002	0.007	0.008	0.005
	(0.001)	(0.001)	(0.001)	(0.007)	(0.007)	(0.007)	(0.007)	(0.008)	(0.008)	(0.006)	(0.006)	(0.006)
Belt countries	0.081	0.092	0.111	−0.875**	−0.949**	−0.843**	−0.793*	−0.857*	−0.766*	−0.215	−0.212	−0.190
	(0.082)	(0.082)	(0.085)	(0.409)	(0.410)	(0.434)	(0.407)	(0.406)	(0.414)	(0.365)	(0.373)	(0.358)
Government ideology	−0.031*	−0.029*	−0.032	0.036	0.021	0.042	0.005	0.008	0.009	0.007	−0.051	0.083
	(0.035)	(0.035)	(0.034)	(0.177)	(0.128)	(0.180)	(0.177)	(0.177)	(0.180)	(0.157)	(0.159)	(0.158)
Observation	118	118	118	118	118	118	118	118	118	118	118	118
*R* ^2^	0.476	0.470	0.466	0.589	0.614	0.612	0.587	0.581	0.580	0.250	0.247	0.245

Notes

Robust standard errors in parentheses. D: day gap between the first case in a country and first case in China. CP: confirmed COVID‐19 cases divided by P, the population in the country *i*. TS: transmission speed. CFR: case fatality ratio. KOF over: index of overall economic globalisation. KOF de facto: globalisation measures actual international flows and activities index. KOF de jure: globalisation measures policies and conditions index. Temperature: average March temperature for each country. Age 65+: population ages 65 and above as a percentage of the total population. Median Age: median age of the population. Distance: distance between China's capital and each countries’ capital. Democracy: democracy index for each country. Belt Countries: 0 for Non‐Belt Countries, 1 for Belt Countries. Government Ideology: 1 for right, 2 for center, 3 for left.

* Statistical significance at 10% level.

** Statistical significance at 5% level.

*** Statistical significance at 1% level.

As found by Puhani (2020) and Wang et al. (2020), temperature differences play a role in the transition of the disease (see Table 2). However, the effect is statistically significant only for the duration to reach a country (lnD): warmer countries got the infection earlier. The age variables (age 65 + and median age) do not affect the day gap D at all, but a larger median age increases the infection rate (CP) and the transmission speed (TS), but reduces both with lower significance for the age 65 + variable. This may simply reflect the different exposure the captured age groups have to the virus due to their activities. A higher median age decreases the case fatality ratio (CFR), but a larger portion of age 65 + people increases CFR. These age effects are consistent with prior expectations that COVID‐19 is more fatal in elderly people (see also Rothan & Byrareddy, 2020). Distance increases the day gap until infection but is insignificant afterwards. We also have assumed that distance has no effect on the case fatality ratio. Democracy exhibits practically no significant estimates throughout, and countries with more left governments face a smaller day gap for transition (D). Belt and Road partner countries of China are not negatively affected in any way: the infection rate (CP) is even lower for those countries, at least in the short‐run period we are studying. The estimates for CP are significant at the 5% level, but the coefficients for day gap for transmission (D) and case fatality ratio (CFR) are not statistically significant at conventional levels.

## 5 CONCLUSIONS

This study provides evidence that globalisation levels of countries affect the transmission speed of the coronavirus, both in terms of first arrival in a country, the infection rate and the fatality ratio. More globalised countries are affected faster and with a larger impact. This has to do with stronger human interactions through travel and migration. The implication is that pandemics can be contained through early measures of temporary inter‐country distancing that focuses on human mobility. This is not an argument against globalisation, however, which makes countries wealthier, more competitive, and more able to invest in health infrastructures and through international collaborations (Dreher, 2006; Potrafke, 2015). The effect can be clearly seen in the lower fatality rates provided in this study. However, the coronavirus crisis should stimulate debates about developing flexible systems to execute appropriate inter‐country distancing measures and determining early indicators to trace future pandemic potentials. Trade policies can be designed to strengthen the effective exchange of disease‐relevant goods and services instead of hindering it.

## Footnotes

^1^
With a world population of 7.8 billion today and 1.8 billion in 1918, the estimated number of 40 million deaths in 1918 corresponds in share terms to 173 million today.

^2^
The disease was first reported on the 31st of December 2019, the global outbreak was reported on the 30th of January 2020, and the pandemic was declared on the 11th of March 2020.

^3^
Also called the case fatality rate.

^4^
For robustness, we checked the relationships between the non‐logarithmic variables. Joint test results and significance of the coefficients of the quadratic versions of the KOF over, KOF de facto, and KOF de jure variants indicate that there are non‐linear relationships in most equations. We, therefore, decided to use the logarithmic specifications.

^5^
Note that with the same set of regressors explaining ln TS, ln D, ln CP, coefficients in ln D and ln CP add up to those estimates for ln TS ("adding up", see Table 2).

^6^
See Gygli, et al. (2019). The index was first developed by Dreher (2006) and revised by Dreher, et al. (2008). See also for an application studying globalization and public employment Gözgör et al. (2019).

## Appendix 1

### The list of countries included in the data set

#### All chosen countries (118 countries)

Afghanistan, Albania, Algeria, Argentina, Armenia, Australia, Austria, Azerbaijan, Bahrain, Bangladesh, Belarus, Belgium, Benin, Bhutan, Bolivia, Bosnia and Herzegovina, Brazil, Bulgaria, Burkina Faso, Cambodia, Cameroon, Canada, Central African Republic, Chile, Congo Republic, Costa Rica, Croatia, Cuba, Cyprus, Czech Republic, Denmark, Dominican Republic, Ecuador, Egypt, Equatorial Guinea, Estonia, Ethiopia, Finland, France, Gabon, Georgia, Germany, Ghana, Greece, Guatemala, Guinea, Guyana, Honduras, Hungary, India, Indonesia, Iran, Iraq, Ireland, Israel, Italy, Jamaica, Japan, Jordan, Kazakhstan, Kenya, Korea, Kuwait, Latvia, Lebanon, Liberia, Lithuania, Malaysia, Mexico, Moldova, Mongolia, Morocco, Namibia, Nepal, Netherlands, New Zealand, Nigeria, Norway, Oman, Pakistan, Panama, Paraguay, Peru, Philippines, Poland, Portugal, Qatar, Romania, Russia, Rwanda, Saudi Arabia, Senegal, Serbia, Seychelles, Singapore, Slovak Republic, Slovenia, Somalia, South Africa, Spain, Sri Lanka, Sudan, Suriname, Sweden, Switzerland, Tanzania, Thailand, Trinidad and Tobago, Tunisia, Turkey, Ukraine, United Arab Emirates, United Kingdom, United States, Uruguay, Uzbekistan, Venezuela, Vietnam.
